# 血液科碳青霉烯耐药革兰阴性菌感染的临床特征及预后危险因素分析

**DOI:** 10.3760/cma.j.issn.0253-2727.2021.07.006

**Published:** 2021-07

**Authors:** 少桢 陈, 晶晶 许, 婷婷 肖, 樱惜 翁, 达兵 陈, 誉 张, 金华 任, 晓峰 骆, 志宏 郑, 晓云 郑, 志哲 陈, 建达 胡, 婷 杨

**Affiliations:** 福建医科大学附属协和医院血液科，福建省血液病学重点实验室，福建省血液病研究所，福州 350001 Fujian Medical University Union Hospital, Fujian Institute of Hematology, Fujian Provincial Key Laboratory of Hematology, Fuzhou 350001, China

**Keywords:** 碳青霉烯耐药革兰阴性杆菌, 耐碳青霉烯类肠杆菌科细菌, 危险因素, Carbapenem-Resistant Organism, Carbapenem-Resistant Enterobacteriaceae, Risk factor

## Abstract

**目的:**

了解血液科碳青霉烯耐药革兰阴性菌（CRO）感染的分布及耐药情况，分析影响CRO感染患者预后的危险因素，为临床CRO感染患者的治疗和预后判断提供依据。

**方法:**

回顾性分析2018年1月至2020年6月血液科确诊的CRO感染患者181例，收集患者的临床和实验室检查等资料，分析影响患者30 d死亡的危险因素。在碳青霉烯类耐药肠杆菌科细菌（CRE）亚组中进一步评价CRE主动筛查的临床意义。

**结果:**

分离出的181株CRO中CRE占47.2％，铜绿假单胞菌占37.0％，肺炎克雷伯菌占32.6％，对碳青霉烯类药物高度耐药，对亚胺培南耐药最低抑菌浓度（MIC）≥16 µg/ml的占76.8％（139/181）。菌株标本来源主要是血样和痰液。CRO和CRE感染患者30 d的全因死亡率分别为（41.4±3.7）％、（44.7±5.4）％。Cox多因素回归分析结果显示：降钙素原水平>0.2 ng/ml及亚胺培南耐药的MIC≥16 µg/ml是CRO感染30 d死亡的独立危险因素。CRE亚组分析结果显示，亚胺培南耐药的MIC≥16 µg/ml为CRE感染30 d死亡的独立危险因素。CRE主动筛查患者的30 d累积生存率高于未筛查患者［（68.0±9.3）％对（50.0±6.5）％，*P*＝0.21］。

**结论:**

高亚胺培南耐药MIC值的致病菌严重影响血液科CRO感染患者预后，病死率高。开展CRE主动筛查有助于早期预防、早期诊断和早期治疗高危患者。

## Abstract (en)

**Objective:**

To study the distribution and drug resistance of Carbapenem-Resistant Organism (CRO) and to analysis the risk factors of CRO 30-day mortality.

**Methods:**

A total of 181 patients with CRO infection diagnosed in Department of Hematology, Fujian Medical University Union Hospital from January 2018 to June 2020 were retrospectively investigated. The clinical and laboratory data of the patients were collected, the prognosis of patients diagnosed with CRO infection in day 30 was followed up, and the risk factors of prognosis were analyzed. The clinical significance of Carbapenem-Resistant Enterobacteriaceae (CRE) active screening was further evaluated in the CRE subgroup.

**Results:**

Among the total of 181 CRO isolates, 47.2％ were CRE, 37.0％ were Pseudomonas aeruginosa, and 32.6％ were Klebsiella pneumoniae, which were highly resistant to carbapenem and had high MIC value, 76.8％ (139/181) of CRO were MIC of imipenem resistance ≥16 µg/ml. The main sources of isolates were blood and sputum. The 30-day all-cause mortality rates of patients with CRO or CRE infection were (41.4±3.7)％ and (44.7±5.4)％, respectively. The COX multivariate regression analysis showed that the level of procalcitonin >0.2 ng/ml and the MIC value of imipenem resistance ≥ 16 µg/ml were independent risk factors for 30-day mortality of CRO infected patients. The CRE subgroup analysis showed that MIC value of imipenem resistance ≥16 µg/ml were independent risk factors for 30-day mortality of CRE infected patients. The 30-day cumulative survival rate of patients with CRE active screening was higher than the patients without CRE active screening [(68.0±9.3)％ *vs* (50.0±6.5)％, *P*＝0.21].

**Conclusion:**

The high MIC value of imipenem resistance isolates seriously affects the prognosis of patients with CRO infection in the hematology department, and the mortality rate was high. CRE active screening is expected for early prevention, early diagnosis, and early treatment for high-risk patients.

碳青霉烯类抗菌药物是临床上治疗耐药革兰阴性菌（G^−^菌）感染的常用药物之一。据2020年全国CHINET细菌耐药监测网的数据显示：临床分离菌株以G^−^菌为主，肺炎克雷伯菌2016-2019年间的检出率呈上升趋势，对碳青霉烯类抗菌药物亚胺培南和美罗培南的耐药率分别从2016年的16.7％和19.7％上升到2019年的25.3％和26.8％。而大肠埃希菌、铜绿假单胞菌、鲍曼不动杆菌的检出率和对碳青霉烯类抗菌药物的耐药率变化不大[1]。碳青霉烯耐药病原菌的出现使G^−^菌感染的治疗更加困难，常导致患者预后不佳[2]。过去十年，全球碳青霉烯耐药革兰阴性菌（CRO）感染的形势日益严峻，已成为一个威胁全球公共卫生健康的突出问题。WHO和美国疾病预防与控制中心（CDC）均把CRO列入首要危险级别[3]。其中碳青霉烯耐药肠杆菌科细菌（CRE）特别值得关注，该菌属可在社区和院内通过质粒介导克隆性广泛传播，同时耐药性不断增加。目前临床推荐以替加环素、黏菌素、磷霉素等为基础的联合治疗方案[4]。但选择单药还是联合治疗、联合用药的最佳组合方案未形成共识，缺少前瞻性随机对照研究的有力证据和高级别推荐[5]。血液病化疗患者易并发细菌感染，以呼吸道感染和血流感染为主，G^−^菌检出率较高[6]–[7]。针对耐药相关的国内外研究结果显示血液病患者并发CRO感染的病死率高于50％，甚至达100％[8]。本研究中，我们分析我院血液科CRO感染患者的临床特征和病原菌分布特点，探索CRO感染患者预后的危险因素，旨在为CRO感染患者的管理和临床诊疗提供参考。

## 病例与方法

一、病例来源收集福建医科大学附属协和医院血液科2018年1月至2020年6月181例次病原学阳性及临床判定确诊为CRO感染患者的临床和实验室检查资料，纳入同一患者同一次入院第一次分离的菌株，剔除同一患者同一次入院分离的同种菌株。

二、病原菌分离、鉴定及药敏分析

病原菌分离及鉴定严格按《全国临床检验操作规程》的相关规定进行，采用法国生物梅里埃公司的全自动微生物分析系统（VITEK2 Compact）进行细菌鉴定和药敏试验，药敏试验结果参考2018年美国临床实验室标准化协会（CLSI）[9]标准判读。质控菌株为大肠埃希菌ATCC25922和铜绿假单胞菌ATCC27853，均由福建省临床检验中心提供。

三、相关定义和判定标准

1. 确诊CRO感染的判定：（1）患者病原学阳性标本为血标本或胸水、腹水、脑脊液等，则为确诊CRO感染。（2）患者病原学阳性标本为痰液、肛周拭子、口腔拭子、咽拭子、中段尿、创面分泌物、粪便等，同种标本或不同标本培养出≥2次同种CRO菌株即为确诊CRO感染；若只培养出一次CRO菌株，临床上合并以下感染体征或影像学证据之一即为确诊CRO感染：①体温>38 °C或体温<36 °C；②寒战、呼吸急促或低血压（排除其他相关可能）；③存在以下任意一种现象：脓尿、尿液浑浊、脓痰、皮肤黏膜红肿热痛、体表破溃并脓性分泌物；④肺部影像学提示肺部有炎性改变；⑤X线或CT提示浆膜腔积液或脓肿（胸腔积液、腹腔积液、肝脓肿、肾脓肿等）。

2. CRE的判定：参考2020年血液恶性肿瘤患者CRE感染的诊治与防控中国专家共识，CRE的定义[10]满足以下任一条件的肠杆菌科细菌：①对任一碳青霉烯类药物耐药［亚胺培南、美罗培南、多利培南的最低抑菌浓度（MIC）≥4 mg/L，或厄他培南MIC≥2 mg/L］；②产碳青霉烯酶；③如果是对亚胺培南天然耐药的细菌（如摩氏摩根菌、变形杆菌属、普罗威登斯菌属），必须对其他碳青霉烯类药物（如美罗培南、厄他培南、多利培南）耐药。

3. CRE主动筛查的方案[10]–[11]：根据中国[11]及WHO[12] CRO感染预防与控制技术指引，CRE主动筛查的人群包括高感染风险的患者（如免疫力低下、入住ICU、血液科病房或移植病房患者等）。CRE主动筛查标本选择：粪便、直肠拭子和肛周拭子作为常规筛查部位。如果患者既往明确有CRE感染，筛查时应再次采集原感染部位的标本送检。CRE主动筛查频率：入院时行首次CRE主动筛查。首次筛查阳性患者，若住院≤30 d，不再进行筛查，实施隔离措施直至出院；若住院>30 d，则每月再行1次筛查，实施隔离措施直至筛查结果阴性。首次筛查阴性患者，则住院期间每周筛查1次。CRE主动筛查流程图详附图1。

四、统计学处理

应用SPSS 25.0软件进行统计学分析，计量资料以均数±标准差表示，计数资料以例数（构成比）表示；危险因素分析：单因素分析采用Logistic回归分析方法，将*P*<0.05的单因素均纳入多因素分析模型；多因素分析采用Cox回归分析方法。运用R语言竞争风险模型进行30 d累积死亡率的竞争风险分析。*P*<0.05为差异具有统计学意义。

## 结果

1. CRO感染患者的一般临床资料：本组数据未有同时存在≥2种［CRE菌株和CRO（CRE除外）菌株］病原菌感染的病例，181例CRO感染患者中，男107例，女74例，平均年龄42岁，平均总住院天数35 d，平均每日住院费用（5428.71±183.78）元。按照WHO 2016分类标准，其中急性髓系白血病（AML）75例，急性淋巴细胞白血病（ALL）34例，淋巴瘤30例，再生障碍性贫血（AA）11例，骨髓增生异常综合征（MDS）10例，多发性骨髓瘤（MM）10例，其他11例，选择移植治疗占30.9％（56/181）。确诊CRO前中位化疗疗程数3（0～29）个，既往3个月碳青霉烯类抗菌药物暴露>14 d的占39.2％（71/181），确诊CRO前粒细胞缺乏（粒缺）时间>7 d占40.9％（71/181）。中位随访328（21～5115）d，1、2、3年累积复发率分别为（14.1±3.4）％、（26.5±4.7）％、（42.8±6.6）％，3年总生存率为（40.1±7.2）％，3年无病生存率为（31.6±4.3）％，感染后30 d的全因死亡率为（41.4±3.7）％。详见附表1。

**表1:** CRO菌株分布和构成比

菌株	株数	构成比（％）
耐碳青霉烯类肠杆菌	85	46.9
肺炎克雷伯菌	59	32.6
大肠埃希菌	18	9.9
阴沟肠杆菌	4	2.2
阿氏肠杆菌	3	1.6
产酸克雷伯菌	1	0.6
耐碳青霉烯类非发酵菌	95	52.5
铜绿假单胞菌	67	37.0
鲍曼不动杆菌	26	14.4
琼氏不动杆菌	2	1.1
解甘露醇罗尔斯顿菌	1	0.6

注：CRO：碳青霉烯耐药革兰阴性菌

2. CRO菌株的分布和耐碳青霉烯类药物MIC分布情况：共分离181株CRO，耐碳青霉烯类非发酵菌和耐碳青霉烯类肠杆菌分别占52.5％（95/181）和46.9％（85/181）；菌株以铜绿假单胞菌为主37.0％（67/181），其次为肺炎克雷伯菌32.6％（59/181）（表1）。根据我院药敏结果，CRO和CRE菌株对碳青霉烯类抗菌药物高度耐药。CRO菌株中对亚胺培南耐药最低抑菌浓度（MIC）≥16 µg/ml的占76.8％（139/181）。CRE菌株中对亚胺培南耐药MIC≥16 µg/ml的占74.1％（63/85）（表2）。

**表2:** 碳青霉烯类抗菌药物不同最低抑菌浓度（MIC）中CRO与CRE菌株分布［株（％）］

组别	株数	MIC（µg/ml）				
		2	4	6	8	≥16
亚胺培南						
CRO	181	2（1.1）	9（5.0）	1（0.5）	30（16.6）	139（76.8）
CRE	85	1（1.2）	6（7.0）	1（1.2）	14（16.5）	63（74.1）
美罗培南						
CRO	74	0	7（9.4）	1（1.4）	17（23.0）	49（66.2）
CRE	18	0	0	1（5.6）	3（16.6）	14（77.8）

注：CRO：碳青霉烯耐药革兰阴性菌；CRE：碳青霉烯耐药肠杆菌科细菌

3. CRO菌株的标本来源分布情况：CRO菌株主要来源于血标本29.8％（54/181）和呼吸道痰液标本28.2％（51/181），其次来源于肛周拭子标本16.6％（30/181）和口腔拭子标本10.5％（19/181）（表3）。

**表3:** CRO菌株标本来源和构成比

标本来源	株数	构成比（％）
血	54	29.8
痰液	51	28.2
肛周拭子	30	16.6
口腔拭子	19	10.5
咽拭子	10	5.5
中段尿	8	4.4
创面分泌物	4	2.2
引流液	3	1.7
粪便	2	1.1

注：CRO：碳青霉烯耐药革兰阴性菌

4. 影响CRO感染30 d预后因素分析：96例CRO（CRE除外）感染和85例CRE感染患者30 d累积死亡率分别为（38.5±5.0）％和（44.7±5.4）％（图1）。

**图1:**
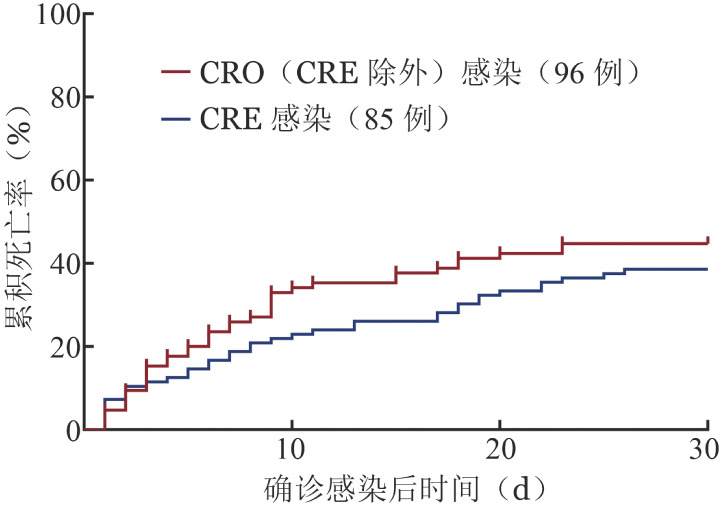
比较CRO（CRE除外）感染和CRE感染患者30 d的累积死亡率 CRO：碳青霉烯耐药革兰阴性菌；CRE：碳青霉烯耐药肠杆菌科细菌

单因素分析见表4，移植与否、确诊前是否达CR、确诊CRO前化疗强度、确诊CRO前住院时间、确诊CRO时降钙素原（PCT）水平、确诊CRO时体温、确诊CRO后抗菌方案和亚胺培南MIC是30 d死亡的影响因素。上述因素纳入多因素模型，结果显示：确诊CRO时PCT>0.2 ng/ml及亚胺培南MIC≥16 µg/ml是CRO感染后30 d死亡的独立危险因素（表4）。

**表4:** 影响181例CRO感染患者30 d死亡的单因素和多因素分析

变量	单因素分析			多因素分析		
	*OR*	95％*CI*	*P*值	*OR*	95％*CI*	*P*值
移植	0.350	0.174～0.704	0.003	0.791	0.338～1.847	0.587
确诊CRO前非CR	2.758	1.281～5.936	0.009	1.533	0.708～3.318	0.278
确诊CRO前化疗强度						
加大剂量	0.390	0.200～0.760	0.006	0.727	0.346～1.528	0.400
减低剂量	0.478	0.042～5.463	0.552	0	0	0.979
常规剂量（参照）	1					
确诊CRO前住院时间>7d	2.336	1.155～4.728	0.018	1.340	0.692～2.598	0.385
确诊CRO时PCT>0.2ng/ml	3.341	1.562～7.148	0.002	2.670	1.324～5.385	0.006
确诊CRO时体温>39.5°C	2.252	1.219～4.160	0.010	1.523	0.895～2.593	0.121
确诊CRO后抗菌治疗方案						
替加环素为基础	1.608	0.599～4.314	0.346	1.378	0.640～2.966	0.413
多黏菌素为基础	1.326	0.526～3.343	0.549	1.727	0.814～3.663	0.155
β内酰胺类为基础	0.368	0.152～0.891	0.027	0.656	0.301～1.429	0.289
单药治疗	0.491	0.174～1.388	0.180	1.028	0.402～2.631	0.954
其他联合	0.553	0.046～6.595	0.639	0	0	0.979
替加环素+多黏菌素为基础（参照）	1					
亚胺培南MIC≥16µg/ml	7.507	2.786～20.230	<0.001	4.197	1.298～13.569	0.017

注：CRO：碳青霉烯耐药革兰阴性菌；CR：完全缓解；PCT：降钙素原；MIC：最低抑菌浓度

85例CRE亚组分析结果见表5，血液病治疗方式、抗菌素治疗方案和亚胺培南耐药的MIC值单因素分析有统计学意义，将单因素分析结果中*P*<0.05的因素纳入多因素分析模型，结果显示亚胺培南耐药的MIC≥16 µg/ml是CRE感染后30 d死亡的独立危险因素。

**表5:** 影响85例CRE感染患者30 d死亡的单因素和多因素分析

变量	单因素分析			多因素分析		
	*OR*	95％*CI*	*P*值	*OR*	95％*CI*	*P*值
移植	0.360	0.136～0.950	0.039	0.577	0.260～1.277	0.174
确诊CRE后抗菌治疗方案						
替加环素为基础	1.579	0.476～5.242	0.456	1.380	0.641～2.972	0.411
多黏菌素为基础	0.758	0.175～3.278	0.711	1.195	0.402～3.550	0.749
β内酰胺类为基础	0.271	0.050～1.480	0.132	0.573	0.132～2.482	0.456
单药治疗	0.189	0.036～0.986	0.048	0.465	0.107～2.022	0.308
其他联合	0.947	0.055～16.309	0.970	0.523	0.069～3.952	0.503
替加环素+多黏菌素为基础（参照）	1					
亚胺培南MIC≥16µg/ml	13.333	2.868～61.996	0.001	6.110	1.416～26.366	0.015

注：CRE：碳青霉烯耐药肠杆菌科细菌；MIC：最低抑菌浓度

5. CRE感染患者确诊前CRE主动筛查的临床意义：本组资料数据共有25例患者确诊前行CRE筛查，其中12例筛查结果阳性。随访该部分患者30 d预后，进行CRE主动筛查和未行CRE主动筛查的患者30 d累积生存率分别为（68.0±9.3）％和（50.0±6.5）％（*P*＝0.21）（图2）；其中CRE主动筛查阳性和阴性的患者30 d累积生存率分别为（75.0±12.5）％和（61.5±13.5）％（*P*＝0.36）（图3）。

**图2:**
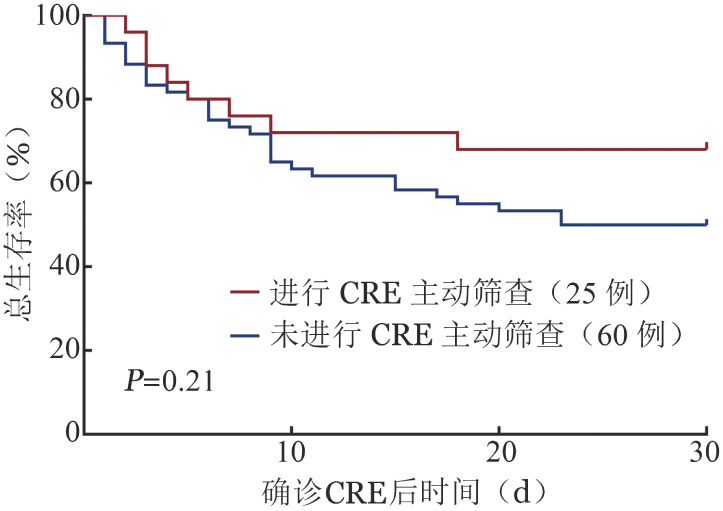
进行CRE主动筛查和未行CRE主动筛查两组患者30 d生存曲线 CRE：碳青霉烯耐药肠杆菌科细菌

**图3:**
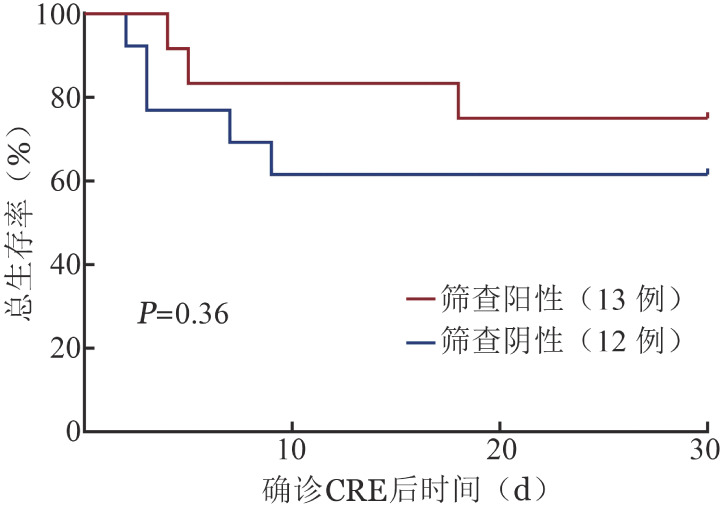
CRE主动筛查阳性和阴性两组患者30 d生存曲线 CRE：碳青霉烯耐药肠杆菌科细菌

## 讨论

本组数据CRO以铜绿假单胞菌为主，占37.0％，高于中东地区圣乔治大学医学中心随访9年数据报道的23.0％[13]，低于国内一组多中心研究报道的59.0％[14]。本组数据中CRE以肺炎克雷伯菌为主，占69.4％。这与2018年全国细菌耐药监测网报道的碳青霉烯类耐药的肺炎克雷伯菌占CRE菌株（8274株）68.6％相近[15]。碳青霉烯类抗菌药物是一种β-内酰胺类抗生素，对产超广谱β-内酰胺酶的革兰阴性菌效果显著[16]。目前运用于临床上的碳青霉烯类抗菌药物有亚胺培南、美罗培南、厄他培南、多利培南等[17]。随着抗菌药物的使用，碳青霉烯类耐药问题随之而来，革兰阴性菌碳青霉烯类耐药机制主要涉及以下3方面：①碳青霉烯酶的产生；②膜孔蛋白的丢失或改变；③外排泵功能的高表达；④药物作用靶点青霉素结合蛋白改变，其中碳青霉烯酶的产生起着重要作用。碳青霉烯酶主要包括以下3类：A类丝氨酸β-内酰胺酶、D类丝氨酸β-内酰胺酶和B类金属β-内酰胺酶[5],[18]。1990年最早报道了来自英国的黏质沙雷菌中发现A类碳青霉烯酶[5],[19]，此后不断报道其他碳青霉烯酶[18]。

本组共有85株CRE，30 d的累积生存率55.3％。黄细莲等[20]的研究发现血液科CRE筛查阳性与患者后续并发CRE感染的病原菌符合率高，起到较好的预警作用，这与本研究结果类似。本组数据中进行CRE主动筛查患者的30 d累积生存率高于未行CRE主动筛查组，但差异无统计学意义，这可能与样本量较小有关。我们认为该结果从一定程度上肯定了CRE主动筛查的重要临床意义，为临床抗生素治疗提供一定依据，早期诊断和治疗患者，降低病死率。因此，我们有必要针对指南[11]–[12]推荐的CRE主动筛查人群积极开展CRE主动筛查工作，并在未来大样本基础上做进一步分析和评价。同时，建议各医疗机构要落实有效的防控措施，包括手卫生、严格的监控流程和上报程序、接触隔离、环境消毒、筛查试验和去定植等[13],[21]–[22]。

血液病患者罹患CRO感染，特别是CRE感染的临床预后差，与基础疾病预后不良、免疫力低下、抗菌药物暴露及住院时间长等可能有关[6],[8],[23]–[25]。本组资料显示CRO患者30 d内全因死亡率高于40％，这可能与该组血液病患者68％粒缺时并发感染有关[26]，也可能是不恰当的初始经验性治疗所致[27]–[28]。CRO菌株易对抗菌药物产生耐药性，临床医师的治疗选择有限，疗效不佳[29]–[30]。目前血液病并发CRO感染患者的最佳抗菌方案并不明确，除了期待新药研发上市和提高病原菌检出的时效性，我们应关注高危患者的预后，早期干预治疗。本研究我们随访181例患者确诊后30 d的生存情况，结果显示亚胺培南MIC≥16 µg/ml是CRO感染死亡的独立危险因素，也是CRE亚组分析的不良预后因素。高亚胺培南MIC的CRE菌株具有高致死率，与该菌株对抗菌药物的强耐药性密切相关，也可能与该种菌株可通过产碳青霉烯酶的可移动遗传元件广泛传播耐药性，导致交叉感染和局部流行有关。目前出台的相关政策尚没有统一的标准，但都强调规范使用抗菌药物，积极推进耐药机制研究和新药开发的进程，多方通力协作，及时采取有效的干预措施，遏制耐药问题的发展[4],[12],[22],[31]。

综上所述，血液病患者并发CRO感染的病原菌以铜绿假单胞菌和肺炎克雷伯菌为主，高亚胺培南MIC的病原菌致死率高，特别是CRE。开展CRE主动筛查有望早期预防、诊断和治疗高危患者。
